# Tropical Abscess of the Liver

**Published:** 1897-09

**Authors:** Edward B. Jackson

**Affiliations:** Houston, Texas


ATLANTA

### Medical and Surgical Journal.

Vol. XIV. SEPTEMBER, 1897. No. 7.

L. B. GRANDY, M.D., MANAGING EDITOR.

M. B. HUTCHINS, M.D., BUSINESS MANAGER.

COLLABORATORS:
A. W. CALHOUN, M.D., LL.D., VIRGIL O. HARDON, M.D., FLOYD W. McRAE, M.D., A. W. STIRLING, M.D., JOSEPH EVE ALLEN, M.D., Augusta, Ga., and HENRY R. SLACK, Ph.M., M.D., LaGrange, Ga.

### ORIGINAL COMMUNICATIONS.

### TROPICAL ABSCESS OF THE LIVER.

By EDWARD B. JACKSON, M.D.,
Houston, Texas.

In probably more than 50 per cent. of all cases there is a history of preceding or succeeding dysentery—usually the dysentery antedates the occurrence of hepatic suppuration, and has been supposed to be in a great number of cases the direct cause. The concurrent manifestations of liver abscess and dysentery show that there is an intimate relationship between the two diseases; and since this is seen in a majority of all cases, it is worthy, as a causal factor, of due consideration in the diagnosis and prognosis of both disorders. The greater number of cases occur in males who inhabit humid, malarious and hot climates. It is easy to see that the functions of the liver are under such circumstances easily and frequently disturbed. Those who are in every way exposed to hardships, poorly fed, clothed and housed, are said to be most easily attacked. We believe that overeating, excessive stimulation, want of proper exercise, and a general indulgence in stimulants and narcotics tend largely to the production of the disease.

Sometimes it may be entirely caused by the impaction of biliary concretions, and occasionally it is the result of injury from without; though it is a fact that a blow over the region of the liver will oftener cause inflammation of the peritoneum. Some authors have claimed that abscess of the liver is rarely a primary affection; that it is the result of the absorption of poisons which are generated in an ulcerous intestine, and that it is, therefore, a pyemic and secondary affection, excepting those cases strictly due to injury or impactions.

The primary anatomical change, whether the cause be infection, injury or biliary embolism, is hyperemia of the hepatic cells, followed by deposits of granular matter, and subsequent degeneration into pus of a pale yellow color and rather thin consistency. The natural tendency is to rapidly encyst or absorb this pus, but occasionally neither condition will obtain, in which event, if left alone, the phlogistic process proceeds along the route of least resistance, breaking down structures all along the way until the peritoneal cavity, stomach, lungs, inguinal region, colon, or exterior of the abdominal wall is punctured for exit. If the matter becomes encysted it ceases to burrow; the inflammation in a measure subsides; the connective tissue wall becomes smoother, and the abscess remains in a cold state until reaction occurs, or it be removed by the surgeon. If absorption occurs immediately upon the production of pus the cavity of its origin is filled in with cicatricial tissue and ultimately repaired virtually perfectly.

The amount of pus which may form in the liver is sometimes enormous—a half-gallon not being an excessive amount. The right lobe is often the seat of mischief, and the abscess discharges oftenest into the thoracic cavity through the right lung. If it be the result of pyemia from intestinal infection, the abscesses are multiple in the beginning; coalescing finally, however, into one large cavity.

If an abscess is seated in the deeper portion of the right lobe, encysted, not materially compressing the bile ducts, not involving the peritoneum in any way, not producing marked systemic disturbances, it will, of course, be impossible to diagnose it short of exploration. Those typical cases which follow recent injury, or duct occlusion from concretions, are easy of recognition. There is the chill; aching limbs, back and head; furred tongue, fever and quick, full pulse. Over the liver there is a stitch of pain; intense tenderness on pressure; heavy, dragging discomfort; the area of dullness on percussion is increased in extent with the inflammatory invasion or amount of pus contained. The temperature is probably only slightly elevated in the morning, going up gradually as the day wears by, reaching 102 F.

There are in a majority of cases shivering rigors probably due to the suppurative process.

If the case be due to impaction of calculi or plugging from any source, jaundice occurs early.

If the abscess be located some distance away from the common bile duct, jaundice will not occur until it has gained such proportions as to seriously impinge upon the outlet of biliary fluid. There are more than 50 per cent. of all cases in which jaundice never occurs. The urine is high colored and full of deposits, and will occasionally show the presence of bile. Perspiration breaks over the patient readily and profusely. After the suppurative process is well established there may be distressing and persistent vomiting of biliary fluid. Bile products are also freely carried off by enteric dejectas, mixed with necrotic blood-clots, and putrid sloughs from intestinal ulcers.

It is impossible to detect fluctuation unless there be a great quantity of pus not deeply seated.

When the abscess burrows through and discharges by the right lung, as it usually does, there is, of course, a degree of pleuro-pneumonic disturbance, and after the pus has been expelled there may be a considerable amount of bloody sputum both from the surfaces of the lung channel and the hepatic pus cavity. These chocolate discharges may be vomited or discharged from the bowels if these respective organs have been burrowed through by the abscess. If the pericardium or peritoneum are entered death occurs speedily from collapse, exhaustion and heart failure. If the abscess encroaches seriously upon the portal vein ascites will be developed.

The duration and cause of the disease depends entirely upon the character of its origin, its location and the constitution of the patient. If the pus be early encysted all prominent symptoms subside and the process may remain latent for a long time until fresh symptoms arise, to free it by burrowing, or until it be evacuated artificially. If the pus be not in the beginning walled off by a limiting membrane all symptoms continue; the patient falls into a septic, typhoid, hectic state, and unless promptly relieved soon dies of exhaustion. Many are naturally relieved by the bursting of the abscess into some one of the cavities heretofore mentioned. Repair is occasionally rapid and usually uneventful after the escape of the pus into the bowels, but is often tedious when the outlet is through the lungs or parietes of the abdomen, unless the primary drainage be of a most perfect nature. The prognosis of hepatic abscess depends upon the extent and character of the lesion, and the measures employed in treatment. If the disease should be the result of pyemia from intestinal sepsis, and form in the liver multiple suppurating processes as it usually does (when thus originating), the case is probably entirely hopeless; for even if the abdominal parietes be carefully incised under rigid asepsis, and the disease treated with correct drainage and antiseptics, there will probably still persist the evening rise of fever, and the typhoid muttering and delirium will not subside; and the patient will sink easily away under the pyemic process in spite of us. It is painful to have to relate that we have several times tried it and met with defeat. The operator can promise positively nothing, save death, for all cases where several pyemic processes are found scattered throughout the hepatic structure. On the other hand, it is said that 50 per cent. of those cases occurring as the result of blows or duct occlusion, when incised and treated antiseptically, quickly recover. Unfortunately all of the author's cases have been the result of pyemic influence from intestinal infection combined with chronic alcoholism, general gluttony and malarial hepatic torpor. We believe, notwithstanding all of our previous failures, that operative measures are entirely justifiable in all cases where the aspirating needle demonstrates the existence of pus, for the case might be found to be of simple origin, and of single cavity in formation, and recover readily; and in any event the patient has some better chance for recovery, and is not, in the event of death, robbed of but a precious few days of life—and those days, were he left to bear them, are necessarily filled with burning pains, fever and general misery.

In the diagnosis of the disease it is essential to exclude (1) hydatid tumor, (2) malignant tumor, (3) thoracic effusion, (4) abscess in abdominal wall, and (5) dropsy of gall-bladder. The first is of slow formation, no pain, no fever, pliable, fluctuates readily and emits the characteristic "purring tremor" of echinococcus. The second, while it is accompanied by rapid wasting and the formation of a tumor, is still not as rapid in growth as hepatic abscess; there is no fever nor so great pain on pressure; the persistent vomiting may, however, very closely simulate that witnessed in suppurative hepatitis. The third is only differentiated by tracing the primary symptoms, which are in the case of thoracic effusions of a pleuro-pneumonic character, and in the disease under consideration are of hepatic character. Later on when the liver abscess is pointing into the lung for evacuation, and therefore superinducing a degree of local pneumonia, it is quite difficult short of aspiration to determine the seat or origin of pus. The fourth is excluded by the absence of vomiting and less general disturbance, and early and free fluctuation. The fifth, enlarged gall-bladder, is pyriform, elastic, fluctuates freely, slow of growth, does not produce such decided general disturbance in any instance, and the symptoms, further than a mere gradual enlargement, are wanting.

The most reliable means of diagnosing hepatic abscess is by introducing the aspirating needle, either in front or behind, and carrying it firmly and gently down into the swelling until the sudden want of resistance of structure announces that the pus cavity is reached. If explored anteriorly the needle should enter at a point just below the last rib and just a fraction to the inner side of a line drawn perpendicular to the nipple. If posterior exploration is made the puncture should be easily below the extremity of the lung and in a direct line with the angle of the scapula.

TREATMENT.

As soon as evidences of hepatitis manifest themselves a large linseed poultice, covering amply the liver area, should be applied quite as hot as can be borne. If there is suspicion of malarial complications, ten grains of the bisulphate of quinine must be given every five hours. If there is accompanying dysentery thirty-grain doses of ipecac should be given every four hours, or in larger and oftener doses, to suit the occasional requirements of the case. Calomel or other preparations of mercury should be withheld, since it has been repeatedly shown that their use augments the activity of the hepatic process. The sulphide of calcium is believed to be of some service in checking the extension of the disease, and aiding in purifying intestinal complications. If the bowels do not partake of the morbid process, chloride of ammonia, twenty grains three times a day, acts excellently. The great object in view is the earliest possible detection and expulsion of pus. In seven days after the beginning of hepatitis there is probably enough good ground for exploratory measures, and if pus is found it must of course be instantly given outlet with free, permanent drainage under antiseptic precautions. If then the abscess is single and simple in origin there may be immediate fall of temperature, cessation of pain, vomiting and general discomfort, and the patient may immediately start up in flesh and strength. Sometimes it happens that after one or two weeks of apparent improvement in cases of pyemic origin the discharge suddenly ceases, pain sets up, fever jumps up and a new pustule has formed. When the original incision is reopened under surgical principles, and the new site incised and pus evacuated, there is apparent improvement for a time. Some cases are said to have recovered after many successive operations for incision of multiple pyemic abscess. We are not, in view of this favorable evidence, discouraged on account of a few personal failures, and expect to continue our treatment by free and early evacuation for all cases. The subjects of tropical abscess are always run down, often have the malarial cachexia, and are occasionally addicted to the regular use of morphine and whisky. They require, from the beginning, supportive treatment, but should never be crowded with stimulants or food beyond the powers of easy digestion and assimilation. Sponge baths over the entire surface of the body with equal parts of alcohol and witch hazel are of the greatest benefit, and large quantities of pure water—not ice cold—cool enough to be refreshing may be admitted. The patient must be protected from weather changes, and his apartments should be so arranged as that the greatest abundance of fresh air and sunlight shall enter and aid in the repair of his wasted power.

